# The value of circulating microRNAs for early diagnosis of B-cell lymphoma: A case-control study on historical samples

**DOI:** 10.1038/s41598-020-66062-1

**Published:** 2020-06-15

**Authors:** Steffen Jørgensen, Isabella Worlewenut Paulsen, Jakob Werner Hansen, Dorte Tholstrup, Christoffer Hother, Erik Sørensen, Mikkel Steen Petersen, Kaspar Rene Nielsen, Klaus Rostgaard, Margit Anita Hørup Larsen, Peter de Nully Brown, Elisabeth Ralfkiær, Keld Mikkelsen Homburg, Henrik Hjalgrim, Christian Erikstrup, Henrik Ullum, Jesper Troelsen, Kirsten Grønbæk, Ole Birger Pedersen

**Affiliations:** 1 Department of Clinical Immunology, Naestved Hospital, Naestved, Denmark; 2 Department of Science and Environment, Roskilde University, Roskilde, Denmark; 3 Department of Hematology, Rigshospitalet, Copenhagen University Hospital, Copenhagen, Denmark; 4 Department of Clinical Immunology, Rigshospitalet, Copenhagen University Hospital, Copenhagen, Denmark; 5 Department of Clinical Immunology, Aarhus University Hospital, Aarhus, Denmark; 6 Department of Clinical Immunology, Aalborg University Hospital, Aalborg, Denmark; 7 Department of Epidemiology Research, Statens Serum Institut, Copenhagen, Denmark; 8 Department of Pathology, Rigshospitalet, Copenhagen University Hospital, Copenhagen, Denmark; 9 Professionshøjskolen Absalon, Naestved, Denmark

**Keywords:** Diagnostic markers, Cancer microenvironment

## Abstract

MicroRNAs are small regulatory RNAs that are deregulated in a wide variety of human cancers, including different types of B-cell lymphoma. Nevertheless, the feasibility of circulating microRNA for early diagnosis of B-cell lymphoma has not been established. To address the possibility of detecting specific circulating microRNAs years before a B-cell lymphoma is diagnosed, we studied the plasma expression of microRNA first in pre-treatment samples from patients with diffuse large B-cell lymphoma and subsequently in repository samples from blood donors who later developed B-cell lymphomas. In addition, we studied the microRNA expression in the diagnostic lymphoma biopsy. The most strongly induced (miR-326) and suppressed (miR-375) plasma microRNA at diagnosis, when compared with healthy blood donors, were also substantially up- or down-regulated in plasma repository samples taken from several months to up to two years before the blood donors were diagnosed with B-cell lymphoma. Importantly, at these time points the donors had no signs of disease and felt healthy enough to donate blood. In conclusion, this first study of plasma microRNA profiles from apparently healthy individuals, taken several years before B-cell lymphoma diagnosis, suggests that plasma microRNA profiles may be predictive of lymphoma development.

## Introduction

B cell lymphomas are the most common hematological cancer in adults with reported annual incidence rates of around 21/100,000 persons in DK^1^. A wide variety of B-cell lymphoma subtypes are recognized by the WHO classification, the most common of which are diffuse large B cell lymphoma (DLBCL) and follicular lymphoma (FL)^2^. Despite significant improvements in outcome, DLBCL as such still carry considerable mortality with an overall three year survival of around 75%^3^. Accordingly, there is a need for biomarkers that are readily applicable in the clinic to facilitate early detection and treatment of patients. Circulating microRNAs may be of potential interest in this regard. MicroRNAs are small RNA strands, consisting of 18 to 23 nucleotides, that regulate the activity of messenger RNA (mRNA)^4^. They are detectable - and have been shown to be quite stable in plasma samples despite the presence of RNases^5,6^. Therefore, microRNAs have attracted considerable attention in recent years, and multiple studies have repeatedly demonstrated that they are dysregulated in virtually all malignancies^7^. In particular, aberrant serum levels of different microRNAs have been reported in patients with various types of lymphoma^8–13^. Only two previous study have studied lymphoma microRNA levels in plasma^14^. Khare *et al*. recently reported an increased level of miR-124 and miR-532-5p and decreased level miR-425, miR-141, miR-145, miR-197, miR-345, miR-424, miR-128 and miR-122 in plasma^15^, whereas Ohyashiki *et al*. previously reported a decrease in the expression level of miR-92a in plasma from individuals diagnosed with Diffuse large B-cell Lymphoma^14^. Despite these promising results, there still lack consensus on which circulating markers are relevant in lymphoma. This is most likely due to difference in sample handling and material. It has been shown that microRNA content in plasma differ based on platelet and lymphocyte counts indicating that platelet lysis and activation is a major bias in these studies^16^. Given this knowledge, it may be straightforward that aberrant microRNA expression might be predictive of lymphoma development; however, to the best of our knowledge this has not previously been thoroughly explored. The aim of this study was therefore to determine if a diagnosis of B cell lymphoma is preceded by aberrant plasma microRNA expression profiles.

## Materials and Methods

### Patients with diffuse large B-cell lymphoma

**Figure 1:**
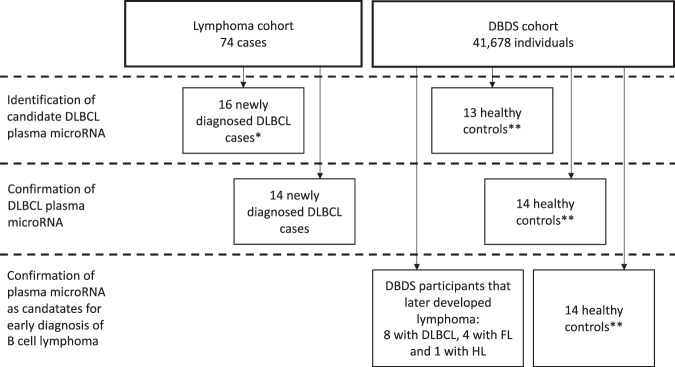
Flow diagram detailing sample collection and study design. We identified candidate plasma microRNAs in treatment naive patients newly diagnosed with DLBCL. The samples were collected as previously described^18^. The deregulated microRNAs were subsequently used for screening of individuals later diagnosed with DLBCL, FL or HL from the DBDS cohort. *The 16 cases were picked because they had previously been examined using expression array on tumor. **The controls could not be matched completely because the DBDS cohort was limited to age 18–67 years. Consequently we selected random controls.

To establish a diagnostic microRNA profile, we retrieved repository samples from 16 patients that in 2006 were diagnosed with diffuse large B-cell lymphoma (DLBCL) according to the WHO classification. On the diagnostic profile sample, we had previously obtained plasma and biopsies before start of treatment^17,18^. Information on clinical presentation, treatment and outcome was obtained from the nation-wide Danish Lymphoma Register (LYFO)^19^. Median age was 66 years (range: 27 to 81 years) and 65% were women. The disease activity of DLBCL cases was high and included 75% with high serum LDH, 62% with extranodal involvement, as well as 75% with Ann Arbor Stage III-IV. Most patients were treated with R-CHOP (60%); however, a few received R-CHOEP (19%) and R-COP (7%). Two patients had relapse of disease and two other patients died within the first year after treatment start (supplementary material Table S1). The microRNA findings from the diagnostic profile was confirmed in a separate cohort of 14 DLBCL patients with a median age of 61 years (range: 44 to 78 years), 50% were women, disease activity high, including 77% with high serum LDH, and 77% with Ann Arbor Stage III-IV (supplementary material Table S2). For overview of the study design and selection of patients see Fig. 1.

### Healthy blood donors, including donors who later developed B cell lymphoma

Healthy controls and apparently healthy individuals, who later developed B-cell lymphoma, were identified in the Danish Blood Donor Study (DBDS). The general design of DBDS has been described elsewhere^20^. By cross linking the first 41,678 DBDS participants with The Danish National Patient Registry (NPR)^21^ 18 blood donors diagnosed with B-cell lymphoma was identified during 125,350 person-years of observation. Diagnosis was subsequently confirmed in 13 of these by histology reports obtained from The Danish National Pathology Data Bank (Patobank)^22^. In total, we identified eight cases of DLBCL, four cases of follicular lymphoma (FL), and one case of Hodgkin lymphoma (HL). The median age of DBDS cases at diagnosis was 48 years (range 28 to 67 years) and 25% were women (see supplementary material Table S3). The date of diagnosis was defined as date of registration in Patobank. Time span between last DBDS sample and diagnosis was 39 to 702 days and between four and nine repository samples were analyzed for each case covering from two to seven years. A total of 41 random DBDS controls were selected, 14 for the screening study, 14 for the confirmation study and 13 as controls for the early biomarker study (supplementary material Table S4). The controls selected for the screening and confirmation study had a median age of 52 years (range 22 to 66 years) and 60% were women. The controls that were compared with DBDS lymphoma cases had a median age of 53 years (range 41 to 64 years) and 38% were women. In six of these controls we also retrieved sequential samples from the plasma repository dating back four years to assess temporal variation in controls.

### MicroRNA assays and in-vitro studies

#### Isolation of plasma RNA and plasma microRNA quantification by RT-PCR

Whole blood was collected in EDTA tubes or EDTA gel tubes from patients, newly sampled blood-donor controls, and DBDS biobank repository samples. The tubes had been centrifuged at 1950g for 10 minutes before freezing. The EDTA tubes from DLBCL cases had been separated and stored at −80 °C as part of a previous study^18^. The DBDS blood donor controls used for the screening and confirmation study were collected in EDTA gel tubes, centrifuged and stored at −80 °C. The DBDS biobank repository samples included separated EDTA gel tubes on both cases and controls that had been stored at −20 °C for up to seven years. After thawing, total RNA was isolated from plasma, using the miRNeasy Serum/Plasma Kit (Qiagen) according to the manufacturer’s instructions. In brief, 300 μL plasma was thawed on ice and centrifuged for 10 min at 10,000 g. Thereafter, 200 μL plasma was transferred to a clean microcentrifuge tube and 750 μL Qiazol containing 1.2 mg MS2 bacteriophage RNA (Roche Applied Sciences) and UniSp 2, 4, 5 spike-in RNA and cel-miR-39 (Exiqon), was added. Total RNA was eluted in 25 μL RNAse-free water.

Initial screening for 172 possible lymphoma-related microRNAs was performed on total RNA from plasma from 14 healthy donors and 16 patients using the Serum/Plasma Focus microRNA PCR Panel (Exiqon) (supplementary material Table S1). Based on these initial results, a targeted screening panel including 11 different microRNAs (Exiqon) was designed (supplementary material Table S5). The panel included the most deregulated microRNAs based on the CT value level and difference between cases and controls. We also included miR-155-5p and miR21-5p because these had previously been reported to be up-regulated in serum^11^. This panel was used to identify lymphoma related microRNAs in repository plasma samples from a confirmatory cohort of 14 DLBCL cases and 14 controls (supplementary material Table S2). The same panel was also used on DBDS donor repository samples from 13 donors that later developed B cell lymphoma and 13 healthy blood donor controls (supplementary material Table S3).

Quantitative real time polymerase chain reaction (RT-PCR) was performed using either the Serum/Plasma Focus microRNA PCR Panel (Exiqon) or the custom panel (Exiqon). Four μl of purified total RNA was reverse transcribed in 20 μL reactions using the Universal cDNA Synthesis Kit II and the UniSP6 spike-in (Exiqon). Conditions for reverse transcription reaction were incubation at 42 °C for 60 minutes followed by five minutes at 95 °C. The resulting cDNA was subsequently diluted 60 times in the ExiLENT SYBR® Green master mix (Exiqon) and assayed in 10 μL reactions according to the manufacturer’s instruction. As inter-plate calibrator we used UniSP3 oligonucleotide. Relative quantitative RT-PCR was performed using the LightCycler 480 and the LightCycler 480 software, version 1.5 (Roche Applied Biosciences). RT-PCR conditions were incubation at 95 °C for 10 minutes followed by 45 cycles of 95 °C for 10 seconds and 60 °C for one minute. Calibration of the different steps was done by adding Cel-miR-39, UniSP2, UniSP3, UniSP4, UniSP5, and UniSP6 according to manufacturer’s instructions.

#### Normalization

Potential reference microRNAs in plasma for RT-PCR were identified using the Genex professional software, version six (Multid). In brief, data from all analyzed Serum/Plasma Focus microRNA PCR Panels were loaded in the Genex software and analyzed using GeNorm^23^ and Normfinder^24^ algorithms. MiR-23a was used for normalization because it was the most stably expressed microRNA. The expression level of this microRNA was acceptable in both cases and controls regardless of sample processing and storage. The intra-assay normalization of microRNA expression levels (threshold cycle (Ct)) were calculated by the ΔCt method subtracting the mean Ct value of the reference microRNAs from the Ct value of each of the other microRNAs in the same assay. The relative expressions of microRNAs in cases compared with controls were estimated by subtracting the mean expression in controls from the individual values in cases (labeled ΔΔCt)^25^. The ΔΔCt was transformed to fold change using the equation 2^−ΔΔCt^. We report fold change. For down-regulated microRNAs the fold change will be presented as negative.

#### Array analysis on primary DLBCL tumors

MicroRNAs were labeled using Genisphere FlashTag HSR according to the manufacturer’s instructions, and analyzed on Affymetrix microRNA version 1.0 arrays. The raw microRNA data files have been deposited at GEO under accession number GSE40239^26^. The resulting raw data were read and normalized in R-3.1.1 using the bioconductor 2.14 package “affy” (1.44.0) using robust multi-array average (RMA). Array data were Poisson distributed.

#### *In-vitro* studies

DLBCL (Toledo (ATCC CRL-2631)) and T-cell (Jurkat (ATCC number TIB-152) cell lines were cultivated with and without stimulation to test whether the up-regulated microRNAs of interest were expressed in these cell types under different conditions. Both cell lines were seeded at a cell density of 1 ×10^6^ cells/ml in T25 culture flasks with 2.5 mL RPMI medium containing 10% heat-inactivated fetal bovine serum (Life Technologies), 100 μg/mL streptomycin and 100 U/mL penicillin, and cultured at 37 °C with 5% CO_2_ for 24 hours before total RNA isolation. The studies were done in triplicates. For stimulation of cells, 50 ng/mL phorbol 12-myristate 13-acetate (PMA) and 1 µg/mL phytohaemagglutinin (PHA) was used. These methods for stimuation has been used previously on Jurkat cell lines^27^ and for Toledo it has been shown that this cell line lacks the B cell receptor^28^. RNA was isolated using miRCURY RNA Isolation Kit - Cell and Plant (Exiqon), treated with DNase I (Roche), and translated to cDNA using miCURY first-strand cDNA synthesis kit (total RNA) (Exiqon). RT-PCR reaction was performed as described above.

### Assessing test-retest variation and sources of bias

To determine the test–retest variation on the qPCR analysis, all samples were analyzed twice and on two different occasions. The inter-assay variation of UniSP3 was low (1.96 × standard deviation (SD) = 0.53 Ct). The intra-assay variation on the other hand depended on the expression level so that it was low at Ct values below 35 (1.96xSD = 0.63 to 0.87 Ct) and higher at Ct values above 35 (1.96 × SD = 1.2 to 1.7 Ct). Consequently, Ct values above 35 were considered as zero or background in all calculations (see supplementary material Fig. S1).

Because it was not feasible to match controls for age and sex, we compared and found no correlation between sex, age and the miR-326, miR-199a-5p and miR-375 values. Also, for the patient samples we estimated whether the normalized levels miR-326, miR-199a-5p and miR-375 correlated with the hemoglobin level, white blood cell count, lymphocyte count, or platelet count (see supplementary material Fig. S2). The Spearman correlations (R^2^) of these microRNAs with blood cell counts were between −0.34 and 0.08 and none of the correlations were statistically significantly different from random.

Some microRNAs are known to correlate with the blood components and degree of hemolysis^29^. As a surrogate marker for hemolysis we used miR-451a levels though this microRNA also have been reported to be associated with other conditions than hemolysis^30,31^. We found more relative hemolysis in the plasma samples from blood donor controls than from patients (supplementary material Fig. S3). This was, however, most likely explained by a lower general level of microRNA in controls compared to cases. There was no statistically significant difference in hemolysis of DBDS samples (cases and controls). Also, DBDS cases and controls were all collected from similarly treated plasma repository samples. Because the miR-451a levels correlated with the grand mean of all microRNAs, indicating that the microRNA quantity depended on degree of hemolysis, we plotted the expression values of microRNAs of interest towards miR-451a to determine whether the microRNAs were influenced by hemolysis (supplementary material Fig. S3). Both the normalized miR-326 and miR-199a-5p levels correlated slightly with degree of hemolysis with an R^2^ of 0.0965 and 0.1817, respectively^29^. MiR-375 did not correlate with miR-451a.

### Statistical analysis

Because almost none of the microRNAs were normally distributed, the statistical difference of plasma microRNA expression between groups was assessed statistically by Mann Whitney U test for independent measurements and by Wilcoxon signed rank test for paired samples. Because cases and controls were not matched for age and sex the analysis was adjusted for age and sex in quantile regressions. Receiver operating curve (ROC) analyses were performed to determine whether the different microRNAs would classify cases correctly both at the last donation before diagnosis of B-cell lymphoma and on the DLBCL screening and confirmation cohorts. Reported statistics from ROC analyses were area under the curve (AUC), sensitivity and specificity. In order to construct a combined prediction model that included all relevant microRNA, we fitted the candidates into a logistic regression with DLBCL as outcome according to the following equation: *ln*(*P*_*DLBCL*_ / 1-*P*_*DLBCL*_) = intercept + estimate 1 × covariate 1 + estimate 2 × covariate 2 + (..).

The number of microRNA in the model was reduced by stepwise exclusion of those microRNAs that were not statistically significant. By isolating P_DLBCL_ in this equation, the probability of DLBCL can be estimated based on the combined biomarkers.

Because of methodological differences, the array data were not directly compared with RT-PCR data. Median and quartile percentage emissions were reported for the microRNAs of interest.

Statistical significance was determined using the Bonferroni correction of the 0.05 significance level so that 0.0003 was the chosen significance level for comparing microRNA using Exiqons plasma/serum Focus panel and 0.003 was the significance level when comparing data using the customized panel. Statistics was done in Genex version 6, STATA, version 13, R studio and Excel.

### Ethics

Oral and written informed consent was obtained from all participants. The Ethical Committees for the Central Denmark (M-20090237), Capital Denmark and Zealand Regions (SJ-570) approved the study. All methods were performed in accordance with the relevant guidelines and regulations. Additionally, the bio bank and research database were approved by the Danish Data Protection Agency (RH30-0444/I-Suite nr. 00922).

## Results

### MicroRNA expression in DLBCL patients

**Figure 2:**
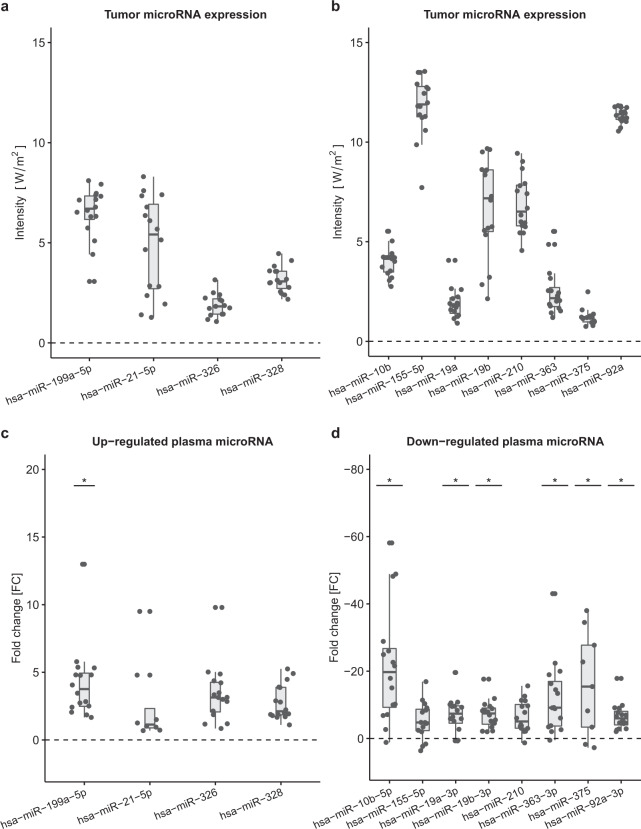
Screening cohort tumor microRNA expression **a**) and **b**). Expression levels in tumor of relevant microRNAs are plotted as intensity (median, 25% and 75% percentiles). Array data were normalized using robust multi-array average. Up-regulated **c**) and Down-regulated **d**) plasma microRNAs plotted as fold change (2^−ΔΔCt^) difference between cases and controls (median, 25% and 75% percentiles). RT-PCR data were normalized using miR-23a. Statistically significant difference (<0.0003) between cases and controls adjusted for age and sex in quantile regressions is marked with a *.

To address whether circulating microRNA could distinguish between healthy blood donors and DLBCL patients at the time of diagnosis, RT-PCR analysis of 172 microRNAs was performed on 16 DLBCL patients from whom there were both plasma and lymphoma tissue available and 14 healthy blood donors using the Serum/Plasma Focus microRNA RT-PCR Panel (Exiqon). MicroRNAs in primary DLBCL tumors from the same patients were analysed using Affymetrix arrays. The microRNA screening in DLBCL patients identified 11 microRNAs that were more than two fold^25^ up- or down-regulated in plasma from patients compared with blood donor controls (Fig. 2 and supplementary Table S5). MiR-199a-5p was the only microRNA that was both up-regulated in plasma (median fold change of 5.9 (range: 1.1–20.2)) and had a high relative expression in DLBCL biopsies. The other microRNAs that were up-regulated in plasma had a relative expression in DLBCL biopsies around the background intensity for the array. With regard to the microRNAs that were highly expressed in DLBCL biopsies e.g. miR-92a-3p and miR-19b-3p, they also had high expressions in plasma but, however, not as high as in healthy blood donor controls and so, compared to controls, the plasma microRNA levels did not especially reflect DLBCL biopsy expression levels.

Most microRNAs that have been reported up-regulated in serum from DLBCL cases in previous studies were not statistically different from controls in the current study. However, we observed down-regulation of plasma microRNAs from the miR-17-92 cluster in DLBCL patients, which is in agreement with a previous study in non-Hodgkin lymphoma^14^ (Fig. 2).

**Figure 3:**
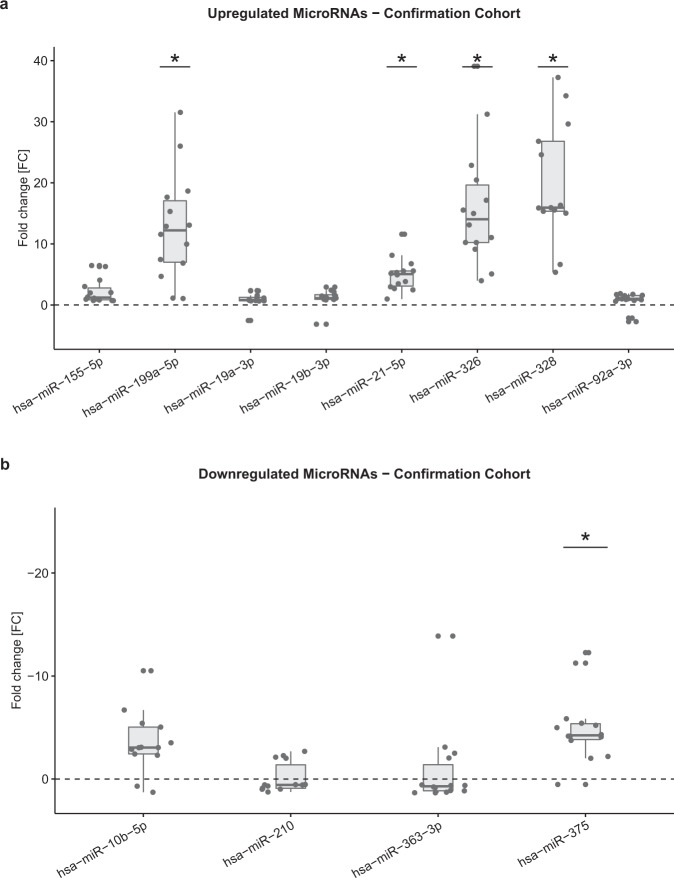
Conformational cohort plasma microRNA expression at diagnosis relative to  plasma microRNA expression in blood donor controls. Up-regulated **a**) and Down-regulated **b**) plasma microRNA plotted as fold change (2^-ΔΔCt^) difference between cases and controls (median, 25% and 75% percentiles). RT-PCR data were normalized using miR-23a. Statistically significant difference (<0.003) between cases and controls adjusted for age and sex in quantile regressions is marked with a *.

For confirmation of the expression levels of the different microRNAs found in the screening study, the same panel with the addition of miR-21-5p and miR-155-5p was tested on plasma from 14 different DLBCL patients and 14 healthy blood donor controls (see: supplementary material Table S2). We included miR-21-5p and miR-155-5p in the confirmation panel because they had been found to be up-regulated in previous studies on serum biomarkers though they did not differ from controls in the screening study. The results of the confirmation are presented in Fig. 3 and supplementary Table S6. In summary, miR-21-5p was statistically significant up-regulated in patients as compared to controls. MiR-155-5p did not differ between cases and controls. Also, the panel narrowed down the number of microRNA that differed between cases and controls. The higher expression level of miR-199a-5p, miR-326 and miR328 in plasma was confirmed in individuals diagnosed with B-cell lymphoma, whereas only the lower expression levels of miR-10b-5p, miR-205-5p, and miR-375 was confirmed on the second B-cell lymphoma samples.

**Figure 4:**
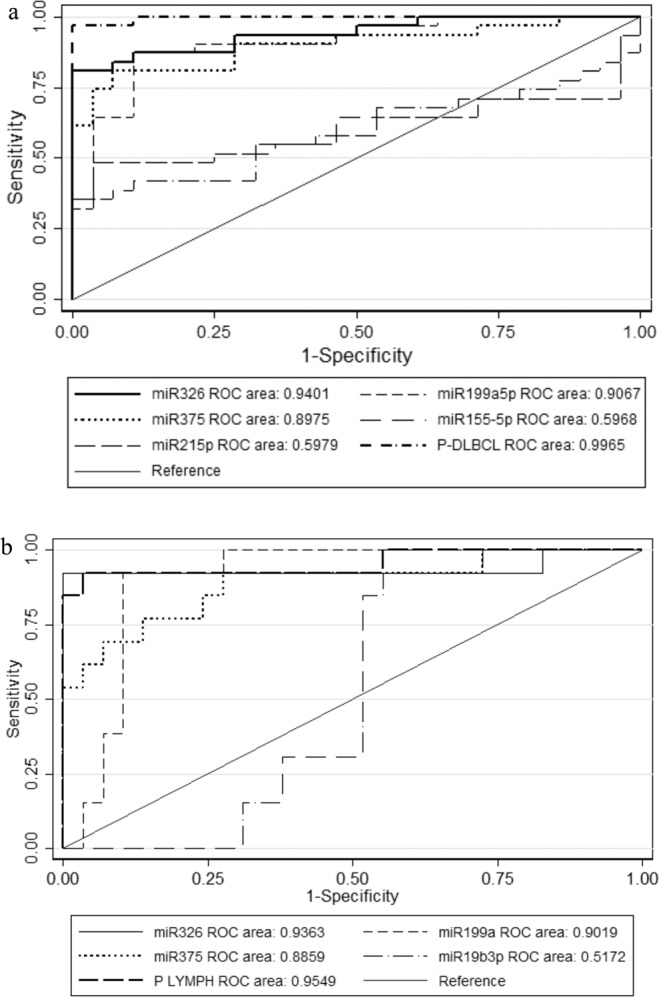
Diagnostic value of plasma microRNA levels. (**a**) ROC of miR-326, miR-199a-5p, miR-375, miR-21-5p, miR-155-5p and the predictive algorithm for DLBCL screening and confirmation cohorts. (**b**) ROC illustrating the sensitivity and 1-specificity of miR-326, miR-199a-5p, and miR-375 in addition to the predictive algorithm for B cell lymphoma compared to healthy controls in last DBDS sample before diagnosis.

We also performed a stepwise logistic regression to identify the most predictive microRNAs based on both the screening and confirmation study (supplementary Table S7). By combining the different microRNA into a linear prediction model based on this logistic regression it was evident that the model that provided the best prediction of DLBCL was a combination of miR326, miR375 and miR19b-3p (R = 0.9124). When we fitted this into the model it resulted in:

P_DLBCL_ = exp(−8.0861 + 1.0053*miR326 + 0.01911*miR375 + 0.1735*miR19b-3p) / (1 + exp(−8.0861 + 1.0053*miR326 + 0.01911*miR375 + 0.1735*miR19b-3p)). The AUC of the algorithm was 0.9965 (Fig. 4a).

### MicroRNA levels in DBDS samples prior to diagnosis of B cell lymphoma

The customized microRNA panel was also tested on DBDS repository samples from 13 blood donors who later developed different types of B cell lymphoma. As controls, we also measured the microRNA levels in 13 blood donor controls with no disease recorded and in yearly samples over a three-and-a-half-year period for six of these. Most microRNAs were not consistently deregulated compared with controls; however, the expression pattern of miR-326, miR-199a-5p, and miR-375 was similar to that observed in DLBCL patients years before diagnosis. For all the other microRNAs in the panel there were either non-significant differences (p values above 0.003) or expression levels above 35 Ct.

### Up-regulated microRNAs

**Figure 5:**
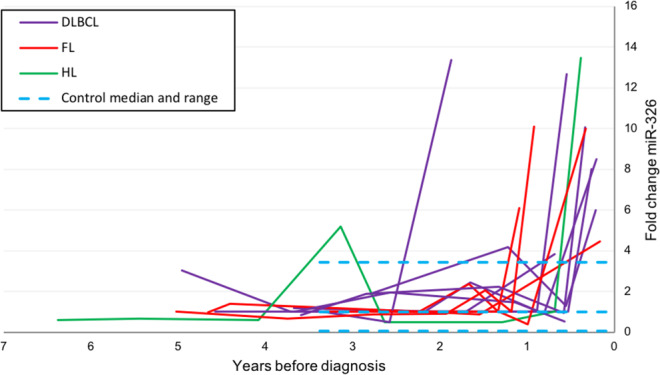
Plasma microRNA expression values (fold change) for miR-326 in repository samples from blood donors diagnosed with B-cell lymphoma. Zero is the time of diagnosis at hospital. The blue dotted lines illustrate the range (variation in expression) in control samples.

In DLBCL patients, the up-regulated microRNAs at the time of diagnosis were miR-328, miR-21-5p, miR-326 and miR-199a-5p. Only miR-326 and miR-199a-5p were also found consistently up-regulated in the last sample the DBDS blood donors donated before they were diagnosed with B-cell lymphoma, at a time where they reported to be healthy and without symptoms of disease. The expression of miR-326 showed a median fold change of 8.2 (range 0.5 to 13.5) at the time of last donation (Fig. 5). In repository samples stored from blood donations obtained during the year that preceded the last donation (N = 20) the relative miR-326 expression was similar to controls (median fold change 1.0 (range: 0.4 – 4.2). The diagnostic value of plasma miR-326 for B-cell lymphoma classification was determined using ROC analysis with an AUC of 0.9363 (Fig. 4b). A cut-off level of two-fold change resulted in a sensitivity and specificity of 92.9 and 97.1, respectively.

By applying the same algorithm as for the screening study we could calculate a combined score for the microRNAs on the last DBDS sample before diagnosis. This combined model had an AUC of 0.9549 which was more or less the same as the AUC for miR-326 (Fig. 4b).

Because circulating miR-326 has not previously been associated with B-cell malignancies, but has been found in T-cell malignancies, we also measured the miR-326 levels *in-vitro* on B- and T-cell lines with and without stimulation to see whether either of these cell types could be the origin of the up-regulated microRNAs. The tests revealed that the miR-326 expression was low in both cell lines regardless of stimulation (supplementary material Table S7).

Plasma miR-199a-5p was also up-regulated several years before the diagnosis of B-cell lymphoma, though not as markedly as for miR-326. The median fold change was 6.9 (range 1.6 to 12.0) and 1.0 (range 0.7 to 13.3) in last sample from DBDS lymphoma patients and in all control samples, respectively. Even though there were some overlapping values among cases and controls the difference was still statistically significant (p < 0.001). Based on the last sample before diagnosis, the ROC analysis revealed that the AUC of miR-199a-5p was 0.9019 (Fig. 4b). Applying a cut-off level of four-fold change correctly classified 85.7% of samples with a sensitivity and specificity of 78.6 and 88.6, respectively.

### Down-regulated microRNAs

**Figure 6:**
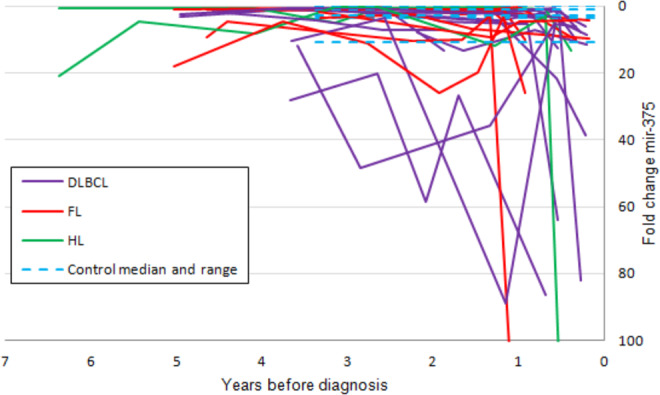
Plasma miR-375 levels before diagnosis. Plasma microRNA expression values (fold change) for miR-375 in repository samples from blood donors diagnosed with B-cell lymphoma. Zero is the time of diagnosis at hospital. The blue dotted lines illustrate the range (variation in expression) in control samples.

The most down-regulated microRNA in DLBCL patients was miR-375. This microRNA was also down-regulated in some but not all DBDS repository samples (Fig. 6). In the last sample from cases vs all control samples, the median relative down-regulation was 23.7 (range 1 to 203.0) and 2.17 (range 0.0026 to 14.27) (p = 0.005), respectively. Down-regulation of miR-375 was present in nine out of thirteen DBDS lymphoma cases in last sample before diagnosis (AUC of miR-375 was 0.8859, Fig. 4b). A miR-375 cut-off of nine-fold change correctly classified 87.5% of samples with a sensitivity and specificity of 69.2 and 94.3, respectively.

## Discussion

The present study showed that aberrant microRNA levels can be demonstrated in plasma for considerable periods of time, i.e. months to years, before diagnosis of B-cell lymphoma. The best markers differentiating B-cell lymphomas from healthy controls (miR-326 and miR-375) have not previously been associated with these diseases.

Whereas the previously reported miR-17-92 cluster was also found to be associated with disease in the present investigation, none of the other previously reported microRNAs i.e. miR-15a, miR-16-1, miR-29c, miR-34a, and miR-210 were selected for further analysis as their expression barely differed between cases and controls. We included miR-21-5p and miR-155-5p in the confirmation cohort, but they did not consistently differ between cases and controls. Despite this, we also tested miR-21-5p in the early blood donor samples. This microRNA was only up-regulated in the last sample before diagnosis in 61% of the donors that developed B cell lymphoma. This difference between our study and the previous ones can presumably be explained by different methodological issues, among others small sample size in combination with low diagnostic value for the microRNAs, use of an internal or external microRNA for normalization, and analysis on plasma rather than serum^29^. Most previous studies selected microRNAs for study based on known up-regulation in lymphoma. The rationale behind this was the hypothesis that the microRNA levels are determined by tumor cell death and apoptosis, and that circulating microRNA reflects tumor burden. In the present investigation we took a different approach by screening plasma samples from newly diagnosed patients to identify markers that may also be valid when testing pre-diagnostic plasma samples for early diagnosis.

Because we had access to plasma samples taken both before diagnosis and at diagnosis in addition to diagnostic lymphoma biopsies on 16 DLBCL cases, the study provided some insight into the dynamics of the aberrant plasma microRNA levels. Both miR-326 and miR199a-5p were up-regulated months to years before diagnosis. For several microRNAs there was a correlation between a high relative expression in DLBCL biopsies and plasma. However, most of these microRNAs were also highly expressed in controls and after correcting for the expression levels in controls only a few plasma microRNA levels (miR-199a-5p and miR-375 (Table 1)) correlated to the level in DLBCL biopsies. All of these findings suggest that the malignant cells are not the source of the differentially expressed microRNAs. Instead, microRNAs may originate from regulatory processes in the microenvironment, such as inflammation. However, the precise role of these circulating microRNAs in B-cell lymphoma remains to be determined.

Interestingly, when pre-diagnostic samples were ordered according to sampling sequence rather than according to time point taken before diagnosis, elevated plasma miR-326 and 199a-5p were almost unanimously observed at the last blood donation before diagnosis. In two blood donors the interval between last donation and diagnosis was more than a year. Because blood donors typically return for donation at regular intervals, it may be speculated that diagnostic delay contributed to the long interval between the observed dysregulation of microRNA in last plasma sample from these two donors and lymphoma diagnosis.

The most up-regulated microRNA in our study and the one with the best diagnostic value compared to healthy controls was miR-326. MiR-326 has previously been shown to be associated with hematopoietic cell types and autoimmune diseases. In a murine model, HOAX9 induces MiR-326 expression during hematopoietic differentiation^32^. It has been shown to inhibit the antiapoptotic Bcl-2 proteins in platelets^33^. It has also been found in cutaneous T-cell lymphomas^34,35^, and other cancers, where it has been reported to be regulating tumor suppressing genes via Nin One Binding Protein (*NOB1*)^36,37^ or Pyruvate Kinase Type M2 (*PKM2*)^38^. In addition, miR-326 have previously been associated with Th17 response in multiple sclerosis^39 and^ it has been implicated in the inhibition of differentiation of B-cells by inhibition of ETS-1 transcription factor and been shown to stimulate the antibody and cytokine production in patients with systemic lupus erythematosis^40^. To further qualify the origin of miR-326, we also measured its expression in Jurkat (T-cell lymphoma) and Toledo (DLBCL) cell lines in triplicates with and without stimulation. The conclusion was that the relative expression of miR-326 was low regardless of stimulation similarly to the expression in tumor. Thus, the origin of miR-326 demonstrated in plasma and the mechanism by which it is associated with B-cell lymphoma remain to be determined.

MiR-199a-5p was also frequently up-regulated in the last sample before diagnosis, but, in contrast to miR-326, there was some fluctuation with up-regulation of this miR in earlier samples. There was, however, also large variation in control samples, so the random variation makes miR-199a-5p a poor choice for a diagnostic test. Intracellular miR-199a-5p can function both as a tumor suppresser as well as an oncogene depending on the cellular context^41^. High expression levels of miR-199a DLBCL cells has been shown to be associated with higher overall survival rate due to increased chemotherapy sensitivity^42^. In the circulation, low levels of plasma miR-199a-5p has been associated with survival in lung cancer^43^ and high levels of serum miR-199a-5p has been associated with inflammatory bowel disease^44^.

The pathogenic importance of down-regulated microRNA in plasma in cancer is a puzzle. However, circulating miR-375 is down-regulated in FL harboring the BCL6 translocation^45^ and several cancers including lung^46^, prostate^47^, pancreatic^48^, and oesophageal cancer^49^. This correlate with the down-regulation in the different tumours, and it has been proposed that miR-375 suppresses several oncogenes such as Astrocyte Elevated Gene 1 (*AEG-1*), Yes-Associated Protein 1 (*YAP1*), Insulin Like Growth Factor 1 (*IGF1R*) and Pyruvate Dehydrogenase Kinase Isoenzyme 1 (*PDK1*)^50^.

The present findings are promising for the use of microRNA for early diagnosis of B-cell lymphoma and indicate that marked changes in plasma microRNA may precede the development of symptoms of incipient disease. However, there are some limitations to our study. First, the data of diagnosis was defined as the time of registration in Patobank which is the date they receive the lymphoma or bone marrow for classification. Consequently, the date of diagnosis depends on how long it takes for patients with symptoms to be referred to a specialist department. Second, we observed higher relative expression of microRNAs related to hemolysis in the control group which might have influenced the finding of up- or down-regulated microRNAs. This was however only the case in initial screening of DLBCL samples. There was no difference in plasma miR451 between DBDS cases and controls. Therefore, hemolysis probably did not influence the overall results of the study. Third, the controls used in the present study were healthy blood donors and they were not matched for age and sex. However, in a clinical setting the relevant control group would be patients referred to hematology clinics for diagnostic work up. Furthermore, the sample size was small which limits the generalizability of our findings. Therefore, larger prospective studies on these biomarkers in B-cell lymphoma patients are warranted.

In conclusion, the present study showed that aberrant microRNA levels can be detected in plasma months to years before diagnosis of B-cell lymphoma. The best markers differentiating B-cell lymphomas from healthy controls (miR-326 and miR-375) have not previously been associated with these diseases. These findings need confirmation in larger settings.

## Supplementary information

Supplementary Material.
