# Supplementary material for: The value of circulating microRNAs for early diagnosis of B-cell lymphoma: A case-control study on historical samples
Source: Sci Rep. 2020 Jun 15;10:9637. doi: 10.1038/s41598-020-66062-1 (PMC7295742; doi:10.1038/s41598-020-66062-1)
Supplement: Supplementary file 1 — Supplementary Material. [file 41598_2020_66062_MOESM1_ESM.docx]

**supplementary material for:**

**The value of circulating microRNAs for early diagnosis of B-cell lymphoma: A case-control study on historical samples**

Steffen Jørgensen, Isabella Worlewenut Paulsen, Jakob Werner Hansen, Dorte Tholstrup, Christoffer Hother, Erik Sørensen, Mikkel Steen Petersen, Kaspar Rene Nielsen, Klaus Rostgaard, Margit Anita Hørup Larsen, Peter de Nully Brown, Elisabeth Ralfkiær,‎ Keld Mikkelsen Homburg, Henrik Hjalgrim, Christian Erikstrup, Henrik Ullum, Jesper Troelsen, Kirsten Grønbæk and Ole Birger Pedersen.

Tables:

| **supplementary Table S1: Characteristics of DLBCL screening cohort** | | | | | | | | | |
| --- | --- | --- | --- | --- | --- | --- | --- | --- | --- |
| Age | Sex | Ann-Arbor | LDH* | Diagnosis year | Sample one † | Treatment | Relapse (Y/N) | Sample two † | Death date † |
| 32 | F | 2 | High | 2006 | 51 | R-CHOP | N | 225 | - |
| 66 | F | 4 | Low | 2006 | 48 | R-CHOP | N | - | 2147 |
| 66 | F | 2 | High | 2006 | 29 | R-CHOP | N | 223 | 1964 |
| 72 | F | 4 | High | 2005 | 41 | R-CHOP | N | 231 | 312 |
| 76 | M | 4 | High | 2006 | 28 | R-CHOP | N | 205 | - |
| 61 | F | 4 | High | 2006 | 13 | R-CHOP | Y | 215 | 1476 |
| 71 | F | 4 | High | 2007 | 21 | R-CHOP | N | 235 | - |
| 81 | M | 2 | Low | 2006 | 44 | R-COP | N | - | 173 |
| 72 | M | 4 | High | 2006 | 28 | R-CHOP | N | - | 2506 |
| 69 | F | 4 | Low | 2007 | 23 | R-CHOP | N | 296 | 2219 |
| 56 | F | 4 | High | 2006 | 35 | CHOEP | N | 214 | - |
| 27 | F | 2 | High | 2006 | 13 | CHOEP | N | 205 | - |
| 64 | F | 4 | High | 2007 | 11 | R-CHOP | Y | 184 | 527 |
| 62 | F | 4 | High | 2006 | 41 | R-CHOP | N | - | - |
| 58 | M | 4 | High | 2006 | 13 | CHOEP | N | 208 | - |
| 69 | M | 4 | Low | 2006 | 38 | R-CHOP | N | 233 | - |
| * Serum-LDH: High or low  † Days after diagnosis. Sample one was taken before start of treatment and sample two was taken at last visit after end of chemotherapy. | | | | | | | | | |

| **supplementary Table S2: Characteristics of DLBCL confirmation cohort** | | | | | | | |
| --- | --- | --- | --- | --- | --- | --- | --- |
| Age | Sex | Ann-Arbor | LDH* | Diagnosis year | Time of sample † | Treatment | Relapse (Y/N) |
| 54 | M | 2 | - | 1999 | 12 | CHOP | Y |
| 64 | M | 2 | Low | 2005 | 50 | R-CHOP | N |
| 62 | F | 3 | Low | 2005 | 48 | R-CHOP | N |
| 50 | M | 2 | High | 2005 | 38 | CHOEP | N |
| 60 | F | 4 | High | 2005 | 10 | R-CHOP | N |
| 60 | M | 4 | High | 2005 | 11 | R-CHOP | Y |
| 78 | M | 3 | High | 2005 | 34 | R-CHOP | N |
| 46 | F | 4 | High | 2005 | 12 | CHOEP | Y |
| 51 | F | 2 | High | 2006 | 6 | CHOEP | N |
| 78 | M | 4 | High | 2006 | 29 | R-CHOP | Y |
| 62 | F | 3 | High | 2006 | 49 | R-CHOP | N |
| 62 | M | 4 | High | 2006 | 32 | R-CHOP | N |
| 67 | F | 4 | High | 2006 | 46 | R-CHOP | N |
| 44 | F | 3 | Low | 2007 | 27 | R-CHOP | Y |
| * Serum-LDH: High or low  † Days after diagnosis. | | | | | | | |

| **supplementary Table S3: Characteristics of DBDS donors that developed B-cell lymphoma** | | | | | | | |
| --- | --- | --- | --- | --- | --- | --- | --- |
| Age at diagnosis | Sex | Diagnosis* | Ann Arbor | LDH † | Time to last DBDS sample ‡ | Number of samples | Observation (years) |
| 39 | F | HL | 3 | High | 141 | 9 | 6 |
| 66 | M | FL gr I | 2 | Low | 272 | 7 | 5 |
| 52 | F | FL gr. II | 1 | Low | 98 | 4 | 3 |
| 61 | M | FL gr. II | 4 | Low | 388 | 6 | 4 |
| 67 | F | FL gr. III | 1 | Low | 39 | 4 | 3 |
| 32 | M | DLBCL | 1 | Low | 219 | 5 | 3 |
| 50 | M | DLBCL | 2 | Low | 70 | 4 | 3 |
| 28 | M | DLBCL | 3 | High | 702 | 4 | 4 |
| 49 | M | DLBCL | 2 | Low | 166 | 4 | 2 |
| 29 | F | DLBCL | 3 | High | 114 | 5 | 5 |
| 41 | M | DLBCL | 1 | Low | 106 | 5 | 3 |
| 32 | M | DLBCL | 3 | High | 237 | 6 | 6 |
| 48 | M | DLBCL | 3 | High | 55 | 4 | 2 |
| * Diagnosis: HL; Hodgkin Lymphoma, FL: Follicular Lymphoma, DLBCL: Diffuse Large B-Cell Lymphoma  † Serum-LDH: High or low  ‡ Days before diagnosis | | | | | | | |

| **supplementary Table S4: Age and sex of random controls** | | | | | | | | |
| --- | --- | --- | --- | --- | --- | --- | --- | --- |
| Screening study ID | Sex | Age | Confirmation study ID | Sex | Age | Early samples ID | Sex | Age |
| 1-1 | F | 53 | 2-1 | M | 45 | 3-1 | M | 41 |
| 1-2 | F | 22 | 2-2 | M | 58 | 3-2 | M | 64 |
| 1-3 | F | 52 | 2-3 | M | 61 | 3-3 | M | 53 |
| 1-4 | F | 51 | 2-4 | M | 48 | 3-4 | F | 42 |
| 1-5 | F | 59 | 2-5 | F | 50 | 3-5 | F | 55 |
| 1-6 | F | 40 | 2-6 | M | 59 | 3-6 | F | 63 |
| 1-7 | M | 34 | 2-7 | M | 50 | 3-7 | M | 50 |
| 1-8 | M | 29 | 2-8 | F | 51 | 3-8 | F | 64 |
| 1-9 | M | 57 | 2-9 | M | 48 | 3-9 | M | 41 |
| 1-10 | F | 61 | 2-10 | F | 50 | 3-10 | M | 48 |
| 1-11 | M | 64 | 2-11 | F | 50 | 3-11 | F | 55 |
| 1-12 | F | 60 | 2-12 | F | 52 | 3-12 | M | 57 |
| 1-13 | F | 63 | 2-13 | F | 54 | 3-13 | M | 45 |
| 1-14 | F | 66 | 2-14 | F | 63 |  |  |  |

| **Supplementary Table s5. Screening cohort tumour microRNA expression and plasma microRNAs expression at diagnosis compared with plasma microRNA expression in blood donor controls** | | | |  |
| --- | --- | --- | --- | --- |
| MicroRNA | Tumour expression (intensity)* | Plasma expression at diagnosis (fold change) † | *P*§ | |
| **Up-regulated microRNAs:** | | | |  |
| miR-199a-5p | 6.7 (6.2 – 7.3) | 3.8 (1-7 – 13.0) | 0.0001 | |
| miR-21-5p | 5.4 (1.3 – 8.3) | 0.6 (-6.8 – 9.5) | 0.8800 | |
| miR-326 | 1.9 (1.4 – 2.2) | 3.1 (0.8 – 9.8) | 0.0014 | |
| miR-328 | 3.1 (2.7 – 3.6) | 2.1 (1.1 – 5.3) | 0.0015 | |
| **Down-regulated microRNAs:** | | | |  |
| miR-10b-5p | 4.1 (3.5 – 4.3) | -19.6 (-58.1 – 1.2) | <0.0001 | |
| miR-155-5p | 11.9 (7.7 – 13.5) | -4.8 (-16.9 – 3.6) | 0.0119 | |
| miR-19a-3p | 1.8 (1.5 – 2.2) | -7.3 (-19.6 – 0.7) | 0.0003 | |
| miR-19b-3p | 7.2 (5.5 – 8.6) | -7.4 (-17.7 – -2.0) | <0.0001 | |
| miR-210 | 6.5 (4.6 – 9.4) | -4.9 (-15.6 – 1.3) | 0.0005 | |
| miR-363-3p | 2.2 (1.7 – 2.7) | -9.2 (-43.1 – -2.0) | 0.0001 | |
| miR-375 | 1.2 (1.0 – 1.3) | -36.2 (-164.0 – 2.7) | <0.0001 | |
| miR-92a-3p | 11.4 (11.1 – 11.7) | -5.9 (-17.9 – -2.1) | <0.0001 | |
| * Expression of microRNAs in micro array on tumour tissue reported as median intensity (25% - 75% percentiles). Array data were normalized using robust multi-array average.  † Median fold change (2^-ΔΔCt^) and range of microRNAs in plasma reported as difference between no cases and controls. RT-PCR data were normalized using miR-23a.  § Probability that the difference between plasma expression at diagnosis and in controls was incidental (Quantile regression adjusted for age and sex). P values below 0.0003 were considered significant. | | | |  |

| **Supplementary Table S6. Confirmation cohort plasma microRNAs at diagnosis compared with blood donor controls** | | |
| --- | --- | --- |
| MicroRNA | Plasma median expression and range at diagnosis * | *P*** |
| **Up-regulated microRNAs:** | | |
| miR-155-5p | 1.2 (-1.4-6.5) | 0.0147 |
| miR-199a-5p | 12.2 (1.1-31.5) | 0.0007 |
| miR-19a-3p | -1.3 (-21.0-2.4) | 1.0000 |
| miR-19b-3p | 1.1 (-15.2-2.9) | 0.2612 |
| miR-21-5p | 5.1 (1-11.6) | 0.0002 |
| miR-326 | 14 (4-39.1) | <0.0001 |
| miR-328 | 16.1 (5.3-68.3) | <0.0001 |
| miR-92a-3p | 1.0 (-2.7-1.9) | 0.7208 |
| **Down-regulated microRNAs:** | | |
| miR-10b-5p | -3.0 (-10.5-2.2) | 0.0259 |
| miR-210 | 0.6 (-2.7-1.3) | 0.2564 |
| miR-363-3p | -1.4 (-13.9-1.3) | 1.0000 |
| miR-375 | -4.2 (-12.3- -1.9) | 0.0001 |
| * Median and range of relative expression of microRNAs in plasma reported as fold change. RT-PCR data were normalized using miR-23a.  ** Probability that the difference between plasma expression at diagnosis and in controls was incidental (Quantile regression adjusted for age and sex). P values below 0.003 were considered significant. | | |

| **supplementary Table S7: Jurkat and Toledo cell lines with and without stimulation*** | | | | | | | | |
| --- | --- | --- | --- | --- | --- | --- | --- | --- |
| microRNA | Toledo with stimulation † | Toledo without stimulation † | Fold change | *P* | Jurkat with stimulation † | Jurkat without stimulation † | Fold change | *P* |
| miR-326 | 13.6 | 14.5 | 1.9 | 0.39 | 8.1 | 9.5 | 2.4 | 0.87 |
| miR-155 | 1.9 | 6.9 | 30.5 | 0.0006 | 7.1 | 12.1 | 31.4 | 0.0007 |
| miR-21-5p | 3.4 | 4.5 | 2.1 | 0.73 | -2.1 | 4.0 | 65.3 | 0.0004 |
| * SNORD38D was used for normalisation. All tests were performed in triplicates.  † Median ΔCt | | | | | | | | |

| **supplementary Table S8. Final model for the logistic regression on screening and confirmation cohorts** | | |
| --- | --- | --- |
| Variable | Odds ratio (95% CI) | p-value |
| miR-375 | 1.0193 (1.0013 – 1.0376) | 0.036 |
| miR-326 | 2.7328 (1.6154 – 4.6232) | <0.0001 |
| miR-19b-3p | 1.1894 (1.0954 – 1.2916) | <0.0001 |
| CI: Confidence interval | | |

| **supplementary Figure S1: Inter-assay variation** | |
| --- | --- |
| a | b |
|  |  |
| c |  |
|  |  |
| a, b and c shows the difference plot of different microRNAs and the 1.96*SD values below and above 35 Ct. | |

| **supplementary Figure S2: Relative hemolysis in cases and controls** | |
| --- | --- |
| a | b |
|  |  |
| a: Ct median, 25 and 75 percentile of miR-451a expression in plasma.  b: Normalized expression (ΔCt) median, 25 and 75 percentile of plasma miR-451a (miR-23a used for normalization). There was higher relative hemolysis (ΔCt) in control samples. This might, however, be a consequence of a lower general microRNA level in controls compared to cases and thus the relative level of red blood cell related microRNAs were higher. | |

| **supplementary Figure S3: Correlation between miR-451a and different microRNAs** | |
| --- | --- |
| a | b |
|  |  |
| c | d |
|  |  |
| MiR-92a-3p (d) has previously been associated with hemolysis and had the strongest correlation with miR-451a. Both miR-326 (a) and miR-199a-5p (b) did also correlate with miR-451a to a lesser degree. Mir-375 (c) did not correlate with miR-451a. | |

| **supplementary Figure S4: Correlation between miR-326 and lymphocytes, leukocytes, platelets and haemoglobin.** | |
| --- | --- |
| a | b |
|  |  |
| c | d |
|  |  |
| Plasma miR-326 shows no correlation with lymphocyte, leukocyte or platelet counts and no correlation with plasma hemoglobin concentration. | |

| **supplementary Figure S5: Correlation between miR-199a and lymphocytes, leukocytes, platelets and haemoglobin.** | |
| --- | --- |
| a | b |
|  |  |
| c | d |
|  |  |
| Plasma miR-199a shows no correlation with lymphocyte, leukocyte or platelet counts and no correlation with plasma hemoglobin concentration. | |

| **supplementary Figure S6: Correlation between miR-375 and lymphocytes, leukocytes, platelets and haemoglobin.** | |
| --- | --- |
| a | b |
|  |  |
| c | d |
|  |  |
| Plasma miR-375 shows no correlation with lymphocyte, leukocyte or platelet counts and no correlation with plasma hemoglobin concentration. | |
